# Interferon Regulatory Factor 1 Polymorphisms Previously Associated with Reduced HIV Susceptibility Have No Effect on HIV Disease Progression

**DOI:** 10.1371/journal.pone.0066253

**Published:** 2013-06-14

**Authors:** Aida Sivro, Lyle R. McKinnon, Hezhao Ji, Joshua Kimani, Walter Jaoko, Francis A. Plummer, Ruey-Chyi Su, T. Blake Ball

**Affiliations:** 1 Department of Medical Microbiology, University of Manitoba, Winnipeg, Manitoba, Canada; 2 Department of Immunology, University of Manitoba, Winnipeg, Manitoba, Canada; 3 Department of Medical Microbiology, University of Nairobi, Nairobi, Kenya; 4 Department of Medicine, University of Toronto, Toronto, Ontario, Canada; 5 National Microbiology Laboratories, Public Health Agency of Canada, Winnipeg, Manitoba, Canada; University of Hawaii Manoa, United States of America

## Abstract

**Introduction:**

Interferon regulatory factor 1 (IRF1) is induced by HIV early in the infection process and serves two functions: transactivation of the HIV-1 genome and thus replication, and eliciting antiviral innate immune responses. We previously described three IRF1 polymorphisms that correlate with reduced IRF1 expression and reduced HIV susceptibility.

**Objective:**

To determine whether IRF1 polymorphisms previously associated with reduced HIV susceptibility play a role in HIV pathogenesis and disease progression in HIV-infected ART-naïve individuals.

**Methods:**

IRF1 genotyping for polymorphisms (619, MS and 6516) was performed by PCR in 847 HIV positive participants from a sex worker cohort in Nairobi, Kenya. Rates of CD4+ T cell decline and viral loads (VL) were analyzed using linear mixed models.

**Results:**

Three polymorphisms in the IRF1, located at 619, microsatellite region and 6516 of the gene, previously associated with decreased susceptibility to HIV infection show no effect on disease progression, either measured by HIV-1 RNA levels or the slopes of CD4 decline before treatment initiation.

**Conclusion:**

Whereas these three polymorphisms in the IRF1 gene protect against HIV-1 acquisition, they appear to exert no discernable effects once infection is established.

## Introduction

Interferon regulatory factor 1 (IRF1) is one of the key players in the HIV infection process, important for early HIV replication as well as initiation of innate antiviral immune responses. HIV replication is controlled at the transcriptional level by a complex interaction between viral and host proteins acting on the viral promoter, the long terminal repeats (LTRs). IRF1 is up-regulated early in HIV infection and subsequently activates HIV LTR transcription even in the absence of the viral transactivator Tat [1], [2]. Deletion of IRF1 binding elements at the 5′ HIV-1 LTR results in impaired promoter activity and decreased replication. IRF1 was recently shown to activate unique antiviral response against viral infections, including HIV-1 [3]. Once infection is established, HIV-1 subverts the IRF1 response enabling viral replication and evasion of the host immune response.

Altered susceptibility to HIV-1 infection has been observed in multiple cohort studies around the world, with a small proportion of Highly Exposed, Seronegative (HESN) individuals remaining uninfected despite repeated exposure [4]. This is the case with a subset of sex workers in Nairobi, Kenya who can be epidemiologically defined as resistant to HIV infection. Several correlates of HIV resistance have been proposed [5]–[10]; amongst the strongest of these are genetic polymorphisms in the IRF1 gene. Peripheral blood mononuclear cells (PBMCs) from patients with protective IRF1 genotypes exhibited significantly lower basal IRF1 expression and reduced responsiveness to IFN-γ stimulation [7]. In addition, cells from individuals with protective IRF1 genotypes show reduced ability to transactivate the HIV-1 LTR when infected with a single-cycle HIV-1 VSVg pseudovirus construct expressing a luciferase reporter gene insert, suggesting a limited ability to support HIV replication [11]. Recently we also demonstrated that HIV-resistant women exhibit an altered transient IRF1 response to exogenous IFN-γ stimulation [12], emphasizing the importance of altered IRF1 expression in the HIV-resistant phenotype.

The relation between the protective genetic polymorphisms and susceptibility to disease acquisition is not absolute. Seroconversion can infrequently occur despite preexisting protective mechanisms, due to behavioral factors correlated with increased viral exposure, immune activation due to presence of other genital infections [13], or other risk-related genetic polymorphisms. While the protective IRF1 polymorphisms restrict HIV replication during the early stages of infection, their impact on disease progression remains unknown. This study examined the role of protective IRF1 polymorphism on disease progression, after the establishment of HIV-1 infection.

## Methods

### Ethics Statement

Informed written consent was obtained from all study participants and the University of Manitoba and Kenya National Hospital Institutional Review Boards approved the study.

### Study Cohort

All participants examined in this study were HIV-infected antiretroviral therapy (ART)-naïve female sex workers from a well-described Kenyan cohort (n = 847) [12], [14]. All of the study participants were sequenced for 3 different IRF1 polymorphisms (619, 179 microsatellite (MS) and 6516) as previously described [7]. Bi-annual follow-up including collection of CD4 data was performed from 1990 onwards. CD4 counts were measured using Becton Dickinson Tritest reagents. Participants were followed for a median of 1,072 days [interquartile range (IQR) 247–2,472 days], and had a median 6 CD4 counts during that period (IQR 2–11). The median age at last visit was 37 (IQR 32–43). Standard of care in Kenya does not include HIV VL; these were analyzed on a randomly selected subset of patients (n = 263). Viral loads were measured using Roche bDNA viral load assay v. 3.0.

### Statistical Analysis

Differences in age and follow-up time between individuals with different IRF-1 genotypes were tested using Kruskal-Wallis test for non-parametric data. Previously, Kaplan – Meier survival analysis was conducted to determine weather polymorphisms in IRF1 played a role in HIV disease progression [7], however this previous preliminary study was limited due to large number of seroincident subjects, an inability to control for CD4 count at enrollment, and a small sample size. Here, we will unequivocally determine the effects of IRF1 polymorphisms on disease progression. Therefore, we analyzed the slope of CD4 decline using unstructured linear mixed models analysis with random effects (slopes and intercept) in a much larger cohort. The dependant variable was the natural log of the CD4+ T cell count, as these have been shown to decline linearly with time [15]. Natural log CD4 counts are comparable to square root CD4 counts, since they are comparable in the ranges studied [16], and advantageous since the interpretation of the estimate is more straightforward. IRF1 genotypes were used as independent predictors of CD4 decline, categorized as three groups: protective haplotypes (619AA, 179+179+, 6516GG); neutral haplotypes (619AC, 179+179−, 6516GT) and haplotypes associated with increased susceptibility (619CC, 179–179−, 6516TT). All three IRF1 loci were analyzed separately, including their interactions with time. Only data for IRF1 619 polymorphism is displayed, as 619A was the primary allele associated with HIV resistance, and the other two polymorphisms are in linkage disequilibrium [7]. Results for the other two polymorphisms were the same unless stated otherwise. Only participants with a baseline CD4>350 were included, as done elsewhere [17], [18], since this is the threshold for ART initiation as recommended by WHO guidelines [19]. For participants where viral load data were available, mixed models analysis was used to examine the relationship between identified IRF1 polymorphisms and viral load, controlling for CD4 count at the time of the VL measure. Statistical analysis was performed using PASW Statistics for Mac version 18.0 (SPSS Inc., Chicago, Illinois, USA).

## Results

### Characteristics of Study Participants

To investigate the role of IRF1 polymorphisms in HIV disease progression, we determined the IRF1 genotypes of 1,492 participants. Approximately 60% of participants were HIV infected (847/1,492). Analysis was performed only on HIV infected participants who had a CD4>350 at baseline (487/847, 57.5%). Of the participants included in the study, 8.9% were homozygous for the protective 619 IRF1 allele (AA), 43.1% were heterozygous (AC) and 48% were homozygous for the non-protective allele (CC). There was no significant difference in age between individuals based on the IRF1 619 genotype (median 42 (AA) vs 36 (AC) vs 38 (CC), p = 0.101, Kruskal Wallis Test). Participants with AA genotype were followed for a median of 2023 days, compared to 1462 and 1746 days for AC and CC genotypes respectively (p = 0.219, Kruskal Wallis Test). The median follow-up for AA genotype was 9 CD4 counts/participant compared to 7.5 for AC and 8 for CC genotypes (p = 0.101, Kruskal Wallis Test). Similar characteristics were observed for the 6516, and the 179 MS, which was expected as these 3 polymorphisms are in strong linkage disequilibrium [12]. Summary of baseline characteristics of the study participants are shown in Table 1. Other socio-demographic characteristics were similar between the compared groups (not shown).

**Table 1:** Baseline characteristics of the study participants (HIV positive with CD4+ T cell count>350, n = 487).

Parameter	IRF1 619 genotype			p-value
	AA	AC	CC	
% Total number (n = 487)	8.9	43.1	48	–
% Female	100	100	100	–
% Kenyan	90	78	69	–
% Tanzanian	10	21	29	–
% Ugandan	–	1	2	–
Age (median, IQR)	42 (34–46.5)	36 (33–43)	38 (33–43)	0.101
Follow-up, days (median, IQR)	2023 (740–3241)	1462 (351.8–3069.3)	1746 (469.5–3261.5)	0.219
CD4 counts at baseline (median, IQR)	508 (407.2–751.5)	590.5 (447.3–794.8)	576 (455–708.5)	0.508
No. of CD4 counts/participant (median, IQR)	9 (3.5–15.5)	7.5 (3.0–13.8)	8 (3.0–14.0)	0.101
Treatment	ART-naïve	ART-naïve	ART-naïve	–
% with VL (n = 263)*	10.6	40	49.4	–
Average log copies/ml (median, IQR)	3.1(1.7–4.0)	3.1(2.0–4.0)	3.0(1.9–4.2)	0.9797

* Standard of care in Kenya does not include HIV VL; these were analyzed on a random subset of patients (total n = 263).

### CD4 Decline is not Affected by IRF1 Polymorphisms

In order to assess the influence of IRF1 polymorphisms on HIV disease progression, we analyzed the association between polymorphisms and the rate of CD4+ T cells decline using linear mixed model analyses (Table 2). As expected, CD4 decline (and thus disease progression over time) was observed in the study population during longitudinal follow-up (p<0.001); however, we did not observe any association between the rate of CD4 decline and specific IRF1 genotypes. We found that the protective IRF1 genotype 619 AA (p = 0.854) and the neutral genotype AC (p = 0.391) did not have a significant difference in CD4 decline compared to those with the non-protective CC genotype (Table 2). A similar lack of association was obtained for the other two polymorphisms (IRF1 6516 GG (p = 0.955), GT (p = 0.436) and IRF1 MS 179+179+ (p = 0.676), 179+179− (p = 0.472) compared to their respective non-protective genotypes). This remained true even if the individuals with CD4 count<350 were included or analyzed separately. Additionally, we performed linear mixed model analysis with baseline CD4 count as a covariate, and addition of this variable did not change the previous analysis of CD4 decline and IRF1 genotype associations (not shown). These results indicate that identified IRF1 polymorphisms do not influence HIV disease progression rate as defined by longitudinal CD4 decline in ART naïve HIV-infected patients.

**Table 2:** Linear mixed models analyses to determine effect IRF1 619 genotypes have on the rate of CD4+T cell decline in Kenyan FSW cohort with baseline CD4count>350.

Baseline CD4 count	Parameter	Estimate (daily)	P value	95% Confidence Interval	
				Lower	Upper
CD4>350	Follow-up (days)	−.000341	.000	−.000410	−.000273
	IRF1 619 = AA*follow-up	–1.573174E-5	.854	−.000184	.000153
	IRF1 619 = AC*follow-up	–4.424301E-5	.391	−.000146	5.728030E-5
	IRF1 619 = CC*follow-up	0^a^	.	.	.

* IRF1 619 genotypes: AA (protective against HIV acquisition); AC, CC (non-protective against HIV acquisition, CC genotype was used as the reference comparison).

### HIV VL is not Affected by IRF1 Polymorphisms

Next we analyzed the association between IRF1 polymorphisms and HIV-1 VL, which is a prognostic marker of HIV-1 disease progression [20], and could potentially associate with differences in IRF1 activity. Linear mixed models analyses were performed in order to account for multiple viral load measures (15 participants had two VL measures and 5 had 3 VL measures at different time points). As expected, VL significantly correlated with the natural log CD4 counts (p = 0.008, Table 3). However, no association was observed between protective and non-protective IRF1 genotypes and HIV VL (p = 0.468 for AA and p = 0.512 for AC compared to CC genotype, Table 3). These data suggest that these particular IRF1 polymorphisms have no apparent effect on driving systemic HIV replication *in vivo* in already infected individuals.

**Table 3:** Differences in VL between 619 IRF1 genotypes, and the influence of logCD4 count in linear mixed model analysis.

Variable*	Estimate	Sig.	95% Confidence Interval	
			Lower bound	Upper bound
IRF1 619 = AA	.181972	.468	−.312139	.676083
IRF1 619 = AC	.107070	.512	−.214074	.428214
IRF1 619 = CC	0^a^	.	.	.
Natural log CD4 count	−.298256	.008	−.519209	−.077304

* IRF1 619 genotypes: AA (protective against HIV acquisition); AC, CC (non-protective against HIV acquisition, CC genotype was used as the reference comparison).

## Discussion

HIV-1 susceptibility and disease progression are influenced by a number of distinct host genetic factors such as IRF1, HLA-B and HLA-C loci and CC chemokine receptor 5 (CCR5) [21]. Previous data from our group suggest that IRF1 polymorphisms play a crucial role during the acquisition of HIV infection. Because PBMCs from individuals with protective IRF1 polymorphisms have decreased IRF1 protein levels, resulting in reduced susceptibility to HIV infection [7], [10], [11], we hypothesized that these same polymorphisms could associate with differences in HIV disease progression. Our data shows that although specific IRF1 polymorphisms associate with decreased susceptibility to HIV infection they show no effect on disease progression, either measured by HIV-1 RNA levels or the slopes of CD4 decline before treatment initiation. Therefore, in HIV+ subjects, the ‘protective’ IRF1 polymorphisms have no prognostic significance on HIV-1 disease progression.

HIV-1 has evolved various mechanisms that evade and modify various aspects of the innate and adaptive immune response enabling the long-term persistence and survival of the virus. As with many other host factors, HIV commandeers IRF1 activity, using it to modify the immune response and perpetuate viral spread. Recently, it has been shown that HIV is able to regulate IRF1 protein levels and function by controlling IRF2 and IRF8 (known IRF1 antagonists) leading to the induction of specific interferon stimulated genes without detectable induction of antiviral Type I or II IFN responses in monocyte-derived dendritic cells [22], [23].

The differential expression of IRF1 in activated versus non-activated target cells may play a role in establishment of a productive HIV infection at different tissue sites. It seems likely that at low activation levels in the mucosal tissues the effect of reduced IRF1 expression due to genetic polymorphisms may be sufficient to prevent initial viral replication and establishment of infection. At this stage, most HIV exposures do not lead to productive infection, as evidenced by a severe genetic bottleneck and small foci on HIV-infected cells [24], [25]. However, the HESN phenotype is relative and some HIV infections still occur. Once HIV infection is established and spreads into an activated systemic lymphatic system HIV-1 may be able to override the protective mechanisms present at the time of exposure, including the IRF1 polymorphisms studied here. In fact there is evidence that HIV infection contributes to IRF1 stimulation and T cell activation thus creating an environment that favors viral replication and spread [26].

In summary, the previously identified IRF1 polymorphisms shown to protect against HIV infection are not associated with the HIV disease progression as defined by CD4 decline and HIV VL. The protective effect of these polymorphisms may not be sufficient to limit HIV replication once initial infection is established. Further studies are required to determine tissue specific cellular and systemic IRF1 levels during an ongoing HIV infection, and determine if IRF1 regulation by other mechanisms may play a role in HIV disease progression.
